# 1410. Methamphetamines and Serious Injection Related Infections: Epidemiology and Outcomes of Alabama’s Drug Crisis

**DOI:** 10.1093/ofid/ofac492.1239

**Published:** 2022-12-15

**Authors:** Myles D Prados, Ellen Eaton, Sera Levy, John R Bassler, Kelly Gagnon, Madison M Jeziorski, Davis Bradford, Leah J Leisch, Li Li

**Affiliations:** University of Alabama Birmingham, Birmingham, Alabama; University of Alabama, Birmingham, Birmingham, Alabama; University of Alabama at Birmingham, Birmingham, Alabama; University of Alabama at Birmingham, Birmingham, Alabama; University of Alabama at Birmingham, Birmingham, Alabama; UAB Heersink School of Medicine, Birmingham, Alabama; University of Alabama at Birmingham School of Medicine, Birmingham, Alabama; University of Alabama at Birmingham, Birmingham, Alabama; University of Alabama at Birmingham, Birmingham, Alabama

## Abstract

**Background:**

The U.S. is facing a steep increase in infectious consequences of intravenous drug use due to the ongoing opioid crisis, surging methamphetamine use, and health care disruptions caused by COVID-19. We hypothesize that the sociodemographic and clinical outcomes of persons who inject drugs (PWID) differ based on their drug of choice (opioids, methamphetamines). Further, we hypothesize that the OUD (opioid use disorder) continuum, including linkage and retention in OUD treatment, will vary depending on co-occurring methamphetamine use. By elucidating differences in these groups, we aim to identify opportunities for interventions along the care continuum.

**Methods:**

This is a retrospective study of hospitalized PWID receiving care at the University of Alabama at Birmingham Hospital for a serious injection related infection (SIRI) between 1/11/2016 and 4/24/2021. We queried the EMR for clinical data and health outcomes. We extracted data on substance use disorder(s), treatments, and linkage to care through review of primary and addiction medicine consultation notes. Using statistical measures of association, we compared demographic factors and clinical outcomes among groups; delineating between those with and without methamphetamine use, and without OUD. When appropriate, additional comparisons were made to detect statistical differences between factors and those with and without methamphetamine use.

**Results:**

Of 370 PWID, 286 had OUD, 94 had OUD and methamphetamine use, and 84 had another substance use disorder. There were significant differences according to drug use disorder with patients with OUD and meth use being mostly White (99%), 42% female, and younger relative to those who use opioids only. Patient directed discharge was most common among those with OUD plus meth use, but death was highest for those with OUD only. The OUD care continuum was similar and alarming for both groups with many gaps in care.

**Figure:**
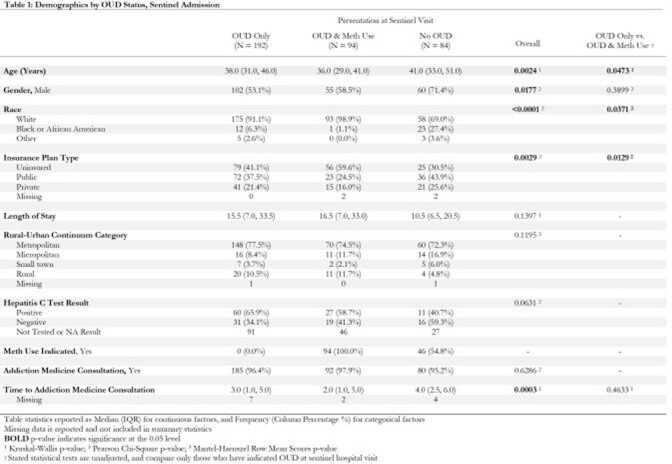

**Figure 1.**
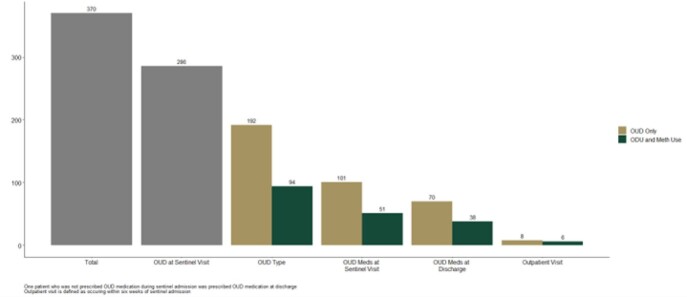
OUD Care continuum for PWID with SIRI for those with and without comorbid meth use disorder

**Conclusion:**

PWID with SIRI are a diverse group with significant differences based on substance of choice, but all experience suboptimal hospital outcomes. There are opportunities to improve linkage and retention across the care continuum, most noticeably outpatient linkage.

**Disclosures:**

**Ellen Eaton, MD, MPH**, Gilead HIV Research Scholar: Grant/Research Support|Gilead HIV research scholar: Grant/Research Support.

